# Why orthotic devices could be of help in the management of Movement Disorders in the young

**DOI:** 10.1186/s12984-018-0466-8

**Published:** 2018-12-14

**Authors:** Lorenzo Garavaglia, Emanuela Pagliano, Giovanni Baranello, Simone Pittaccio

**Affiliations:** 1 Institute of Condensed Matter Chemistry and Technologies for Energy, National Research Council of Italy (CNR-ICMATE), via Previati 1/E, 23900 Lecco, Italy; 2 Developmental Neurology Unit, C. Besta Neurological Institute Foundation, Milan, Italy

**Keywords:** Movement Disorders, Dynamic orthosis, Rehabilitation, Neurophysiological models, Functional materials, Dyskinesia, Dystonia

## Abstract

**Background:**

Movement Disorders (MD) are a class of disease that impair the daily activities of patients, conditioning their sensorimotor, cognitive and behavioural capabilities. Nowadays, the general management of patients with MD is based on rehabilitation, pharmacological treatments, surgery, and traditional splints. Although some attempts have been made to devise specific orthoses for the rehabilitation of patients affected by MD, especially the younger ones, those devices have received limited attention.

**Main body:**

This paper will principally discuss the case of upper limb rehabilitation in Childhood Dyskinesia (CD), a complex motor disease that affects paediatric patients. Through a critical review of the present solutions and a discussion about the neurophysiological characteristics of the disease, the study will lead to the formulation of desirable features of a possible new upper-limb orthosis.

**Conclusions:**

Design principles will be derived to provide specialised orthoses for the dynamic control of posture and the stabilisation of voluntary movements: those include using biomechanical actions and enhanced proprioception to support the sensorimotor rehabilitation of the children affected by CD. A similar approach could be advantageously applied in other MD-related conditions, especially with hyperkinetic and/or hypertonic traits.

## Background

The present paper, taking the move from the peculiar neurophysiological and phenomenological characteristics of Movement Disorders (MD), especially their dynamic and changing nature [1], tries to approach the problem of patients’ functional recovery from the perspective of Biomedical Engineering, i.e. through system analysis and conceptual design. The purpose is to discuss a framework solution for orthotics, specifically dedicated to dyskinetic young patients, the aim being to manage their process of rehabilitation and functional recovery better than it can be done with standard systems in use today. We decided to consider, in particular, Childhood Dyskinesia (CD), a disorder that hinders the motor abilities of the subjects causing difficulties in movement control and the insurgence of unwanted gestures or poses [2–4]. This disorder affects posture, gait and the child’s manual skills, which are essential to develop autonomies, be educated, learn new abilities and conduct a rich psychological and social life [5]. The dyskinetic children must be supported in improving their abilities to play, feed themselves, study and train in manual activities. Solutions adopted over the years, such as pharmacological, rehabilitative treatments, surgical interventions, together with (mostly traditional) orthotic devices, helped improve the overall conditions of the patients. Those same methods often fail to restore functional abilities, due to the complexity of the pathophysiological substrates, so the introduction of new non-invasive methods could be advantageous [6].

What are the weaker points of the present orthotic solutions used in the management of Childhood Dyskinesia? Why is the use of wearable devices so limited in practice and often disregarded in therapeutic designs? How can devices be improved and made more specific for this type of disease?

We believe that a reason can be found in the poor coherence of orthotics with the neurophysiology of MD, as most of the available devices for children are optimised for spastic syndromes (and especially for the lower limb [7]) or orthopaedic applications. In order to be effective in supporting the rehabilitation of dystonic and dyskinetic children, a wearable device should be designed according to the main characteristics of the underlying disorder; in addition, it should be modular or adaptable enough to be functionally personalised to the varying individual patient’s requirements. This paper addresses in particular the former aspect, i.e. the general design principles, while the personalisation issues will be barely touched upon, and left for a future work.

The focus of the review will be on the *upper-limb* orthotics, even though some background information will be derived from the general literature, including orthotics for different body districts. The first part of the paper presents the target population by a definition of the main neurological characteristics of CD; the available treatment solutions will be reviewed; then some recent neurophysiological results will be discussed in order to highlight the most relevant fundamental characters of the disease that should be addressed by innovative devices; finally, a discussion of the present solutions in the light of the neurophysiological findings will lead to a list of desirable technical requirements for wearable upper-limb orthotics dedicated to Childhood Dyskinesia.

## Methods

The present review is not a systematic review. In the relative scarcity of scientific information about the use of specialised orthotics for hyperkinetic MD’s, we carried out an extensive search on the topic using scientific and general browsers, by combining search terms such as “orthosis” or “orthotics”, or “splint” or “brace” or “casting”, with “rigid” or “soft” or “dynamic”, and with “dyskinesia”, “dystonia”, “hyperkinetic”, “cerebral palsy”, “movement disorders”. We tried to be inclusive, every time the source presented a sufficiently-well described solution. Since the focus of the review is on the upper limb, we excluded papers that concentrated only on the lower limbs or gait.

### Definition of Childhood Dyskinesia

Dyskinesias are hyperkinetic movement disorders [6]. In paediatric patients, dyskinesia often presents mixed MD components [4] and identification of some symptoms can be challenging [4]. Practically, patients with Childhood Dyskinesia are often classified by the predominant Movement Disorder, which affects them, with a listing of secondary disorders [8, 9]. Most often dyskinetic children show a mixed hypertonia, with components of spasticity and dystonia [10]. It actually appears that dystonia is a very relevant component in all forms of Childhood Dyskinesia. In fact, common observation of dystonia suggests that it can cause hyperkinetic movements as well as hypertonic movements [11].

Dystonia is characterised by sustained or intermittent muscle contractions that cause abnormal, often repetitive movements, postures, or both [11, 12]. It is possible that dystonia itself has a role in causing hyperkinetic movements, i.e. by inserting dystonic postures that interfere with the voluntary movements. If multiple brief dystonic postures are inserted, variable, jerky and tremulous movements may arise. On the other hand, if the inserted dystonic postures are sustained, movement can become hindered and slower than expected [6]. Other components of Childhood Dyskinesia include more dynamic movement disorders and, in practice, many children with hyperkinetic Movement Disorders have a combination of chorea, athetosis or ballism; dystonia, tics or myoclonus may also be present [2].

Childhood Dyskinesia is mainly seen in Cerebral Palsy (CP) syndromes correlated with extrapyramidal lesions showing increased involuntary movements including dystonia, chorea, athetosis and tremor [2].

### Present treatments and solutions

The focus of the present section is on rehabilitation (including physiotherapy) and orthotics, while pharmacological and surgical treatments [1, 11, 13] will not be discussed.

#### Rehabilitation

Physical therapy integrated with occupational therapies is the basis of CD rehabilitation. Central to this approach is the idea that motor symptoms should not be seen as the sole object of treatment, but their control should be related to the execution of fundamental functions like walking, manipulating objects, communicating, being independent in activities of daily living, and also enjoying high quality of life and social participation.

Advances in the comprehension of underlying mechanisms provided by Neuroscience studies, and the consequent development of new rehabilitation models have indeed confirmed the need for a multidimensional therapeutic approach to children with Movement Disorders, conceiving function as a perceptual-motor-cognitive process: neurophysiological studies on the mental representation of movement, modern functional neuroimaging techniques and the development of cognitive psychology have all contributed to increasing our knowledge about the potential effect of rehabilitation on neural re-organisation processes [14, 15]. The role of the proprioceptive system has been stressed in many different physiotherapeutic approaches [5, 16, 17] including recent motor learning models [14, 18]: this system is recognised as crucial in action planning, motor program execution, and also in execution feed-back.

#### Orthotics

Well-fitted braces are designed primarily *to improve posture and prevent contractures in dystonia* [1, 13, 19]. Correspondingly, most emphasis in the use of the orthotics for cerebral palsy has often been placed on avoiding deformities rather than favouring functional rehabilitation [7]. On the other hand, it has been suggested that in childhood dystonia, soft or semi-rigid braces in particular could help the *functional positioning during movement, containing the joint and giving proprioceptive information* [19].

While there is substantial evidence confirming that ankle foot orthoses can improve gait efficiency in ambulant children, little high quality evidence exists to support the use of orthoses for the hip, spine or upper limb. Where the evidence for orthosis use was not compelling, consensus has been anyway reached on recommendations for orthotic intervention [7].

**Fig. 1:**
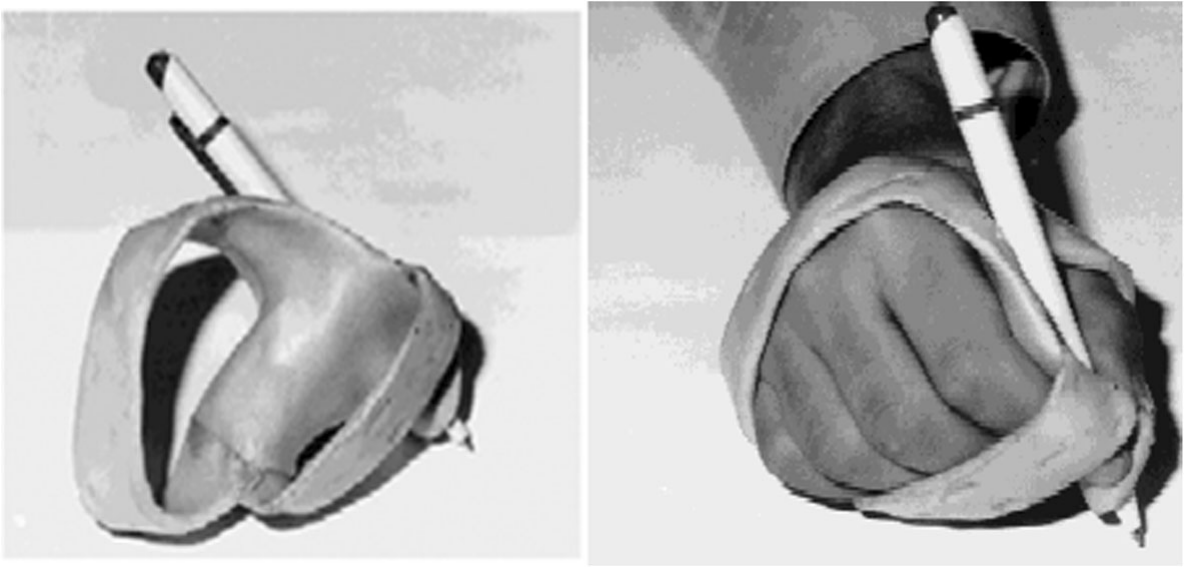
*On the left*, the picture shows a rigid hand orthosis designed and constructed for a subject; on the right, the same device worn by a subject affected by writer’s cramp (courtesy of Taş [69])

Here will be presented a number of wearable devices found in the literature, which have been divided, for our purposes, in the following categories: casting and rigid orthoses (Table 1, Fig. 1), soft orthoses (Table 2) and devices providing stimulation (Table 3).

**Table 1:** casting and rigid orthoses

Type of publication	Aims and summary	Approach and Sample size	Conclusions	Ref.
Scientific paper	Studying the effect of intensive neurodevelopmental therapy (NDT) and upper-extremity inhibitive casting, separately or in combination, on hand function, quality of upper-extremity movement and range of motion.	Clinical study. Comparison of two different intensities of NDT with and without bivalved casts. 73 children with cerebral palsy. Age: 18 months to 8 years	There was no significant difference between intensive or regular therapy and casting or no casting for hand function, between intensive and regular NDT, or between intensive NDT plus casting and the other groups for quality of movement and range of motion. Casting led to increased quality of movement and wrist extension after 6 months. Casting with NDT improved the quality of upper-extremity movement and range of motion. There appear to be no immediate benefits from intensive therapy alone.	[70]
Scientific paper	Studying the effect of neurodevelopmental therapy (NDT) and upper-extremity inhibitive casting, separately or in combination, with occupational therapy (OT), on hand function.	Clinical study. Cross-over trial between intensive NDT with casting, and OT. 50 children with cerebral palsy. Age: 18 months to 4 years	Analysis of the outcomes revealed no significant differences in hand function, quality of upper-extremity movement, or parents’ perception of hand-function performance between the two treatment groups. There does not appear to be any beneficial effect of an increased amount of therapy for the children in this study.	[71]
Scientific review	Reviewing the treatment of hyperkinetic movement disorders. In describing different approaches to the treatment of various movement disorders, the paper includes a statement about casting for dystonia.	Review of the literature.	Immobilisation can actually exacerbate or even precipitate dystonia, as is the case in peripherally induced dystonia.	[1]
Book chapter	Describing different devices employed especially to avoid deformities, block the function, and prevent the beginning of the chain created as a functional pattern.	Summary of rehabilitation strategies for dystonia in children.	Traditional splints or rigid orthoses, made of relatively thick thermoplastic, can provide rigidity, position and stretch the muscles in patients with increased muscle tone. To provide some positive effects, the devices should be designed for each patient, due to the particular needs required by every clinical picture.	[19]
Scientific review	Reviewing the evidence on the effectiveness of using upper and lower limb casting or orthoses in children with cerebral palsy.	Review of the literature.	Further investigation are needed to prove some positive effects, the devices should be designed for each patient, due to the particular needs required by every clinical picture.	[20]
Scientific paper	Evaluating modification in writing ability by immobilisation in patients with hand dystonia. Static hand orthosis was used to support and immobilise the segments of the hand interfering with writing (thumb, fingers, or wrist), and allowing the proximal large muscles to control the writing movements (Fig. 1).	Clinical single-cases study. 5 adults patients affected by hand dystonia. Age: 35 to 59 years old.	Results demonstrated an improvement in writing ability of patients while using the hand orthosis. Authors recommend the application of the hand orthosis in association with other therapies to overcome a disability that handicaps the patient’s daily life and vocation.	[69]
Scientific paper	Evaluating the combined use of an orthosis and occupational therapy. An elbow immobiliser has been prescribed during the application of an occupational therapy programme, to modify the hand function in children with athetoid CP. The device restricted the unwanted movement around the proximal joints (shoulder and elbow joints).	Clinical study. 40 children with athetoid cerebral palsy. Age: 6.51 ± 0.97 years.	Wearing the elbow immobiliser during the application of occupational therapy program has a potential benefits to improve the hand function athetoid CP patients, through controlling the involuntary movement and allowing good performance during fine motor training for longer time and in an effective way. The significant improvement noticed in hand function in the study group vs. controls (that only did the occupational routine) may be due to reduced distal involuntary movement obtained by wearing the elbow immobiliser during training of fine motor skills.	[46]
Scientific paper	Evaluation of a hand splint. Therapy involved immobilisation by splint(s) of 1+ of the digits other than the focal dystonic finger and Repetitive exercises in coordination with 1+ of the other digits.	Clinical single-cases study. 5 adults patients affected by focal hand dystonia.	Immobilising the unaffected limb can help exercise more the impaired limb Positive results in 3 out of 5 musicians with focal hand dystonia after such kind of therapy.	[72]
Scientific review	Reviewing the effects of constraint-induced therapy. Constraint-induced movement therapy has been applied during the rehabilitation of patients with spasticity after stroke to limit non-dystonic segments and to exercise the affected ones.	Review of the literature.	Unclear benefit using constraint-induced therapy, in the treatment of dystonia.	[1]
Scientific paper	Evaluating the effect of movement restriction in cerebral palsy. The research hypothesis was that restriction of the less-involved hand with a resting splint would result in increased use of the more-involved hand in a child with spastic cerebral palsy.	Clinical single-cases study. One 2-year-old girl with greater involvement of the right side.	An improvement in quality, quantity, and variety of use of the more-involved extremity after splinting, with some continuing improvement. Results for a single-case; necessity to enlarge the population tested.	[73]
Systematic review	Describing systematically the best available intervention evidence for children with cerebral palsy (CP).	Systematic review of systematic reviews.	The lack of certain efficacy evidence for large proportions of the interventions in use within standard care is a problem for people with CP, healthcare providers, purchasers of healthcare and funders. More research using rigorous designs is urgently needed as CP is the most common physical disability of childhood with a life-long impact.	[17]
Review article	Investigating treatments and current evidence to improve upper limb outcomes and goal attainment.	Review of the literature.	Some results have been obtained on larger cohort of patients even if affected by mainly spastic forms.	[74]
Scientific paper	Evaluating the effect of segment immobilisation on motor scheme recovery. Casting or immobilisation by an orthosis has been suggested also as a treatment aiming to deprive the dystonic segments of motion and sensation, which could help patients reset lost motor schemes.	Clinical study. 8 patients with idiopathic occupational focal dystonia of the upper limb, the dystonic forearm and hand were immobilised with a plastic splint.	Limb immobilisation can be a simple, effective, safe, and inexpensive treatment for focal occupational upper-limb dystonia.	[45]

**Table 2:** Soft orthoses and orthotic suits

Type of publication	Aims and summary	Approach and Sample size	Conclusions	Ref.
Book chapter	Describing different devices employed especially to avoid deformities, block the function, and prevent the beginning of the chain created as a functional pattern.	Summary of rehabilitation strategies for dystonia in children.	Splints made of soft, semi-rigid or combined materials can be useful in supporting functional position during movement, containing the joint and giving proprioceptive information.	[19]
Scientific paper	Evaluating Lycra garments: (1) changes in functional daily skills, (2) changes in posture and movement of the upper body, (3) client/families’ assessment of their child’s responses and function/behavioural changes.	Clinical study. 12 children, with athetosis, ataxia, and spasticity.	The functional benefit of Lycra garments for children with CP is mainly due to improvements in proximal stability, but this should be weighed against the inconvenience and loss of independence.	[75]
Scientific paper	Reporting on the orthotic management of cerebral palsy. In particular, Lycra garments are designed and used to allow some voluntary movements or at least preserve and favour residual motion.	Report about the recommendations from a consensus conference.	Effectiveness of Lycra soft garments to improve function is not established, and should be evaluated carefully.	[7]
Scientific review	Investigating if Lycra garments could improve function and posture in children with cerebral palsy.	Systematic review of the literature.	Many of the studies considered, include children with different types of cerebral palsy, but do not use objective outcome measures. Some positive results should be weighted with discomfort, loss of independence and costs of the garments. For this kind of treatment, individual assessment and prescription is essential.	[47]
Scientific paper	Evaluating the effects of the Adeli suit (various versions).	Clinical study. 45 patients with acute cerebrovascular lesions (ACVL) and 10 patients with hyperkinetic syndromes.	Data obtained for the hyperkinetic group are particularly preliminary in nature. Signs of hyperkinesia were reduced. Positive effects lasted 1.5–3 h, and hyperkinesia then returned.	[76]
Scientific paper	Comparing the efficacy of Adeli suit treatment (AST) with neurodevelopmental treatment (NDT) in children with cerebral palsy (CP).	Clinical study. 24 children with CP Age: 6 to 12 years.	Improvement of efficacy index for children with higher levels of motor function, without the gain of additional gross motor skills, as reflected by a reduced metabolic cost of external work. Not evident the retention in comparison to other treatments.	[77]
Scientific paper	Examining the effects of wearing a therapeutic suit during an intensive therapy program on motor function among children with cerebral palsy.	Clinical study. 20 children were randomized to an experimental (Therasuit) or a control (control suit) group and participated in an intensive therapy program.	Children wearing the Therasuit during an intensive therapy program did not demonstrate improved motor function compared with those wearing a control suit during the same program.	[78]
Scientific review	Evaluating the available evidence on the effects of interventions based on the use of therapeutic suits in the treatment of impairments and functional limitations of children with cerebral palsy.	Systematic review of the literature	The low quality of evidence suggests caution in recommending the use of these therapeutic suits.	[48]
Systematic review	Describing systematically the best available intervention evidence for children with cerebral palsy (CP).	Systematic review of systematic reviews.	Conflicting evidence. One trial suggests positive effect the other suggest no benefits in using Therasuits for the improvement of gross motor function.	[17]

**Table 3:** Devices providing stimulation

Type of publication	Aims and summary	Approach and Sample size	Conclusions	Ref.
Scientific paper	Evaluating a portable device including electromyography (EMG) and vibration feedback of muscle activity for providing a sensory stimulation (peripherally) on the affected limb. The hypothesis is that additional proprioceptive feedback, could help facilitate motor control.	Preclinical trial. 11 children, with cerebral palsy or acquired static brain injury. Age: 6 to 16 years	The ability to voluntarily and selectively control the activation of a target muscle at different levels of the upper limb kinematic chain could be improved by means of a EMG-based vibrotactile biofeedback device placed on the skin over that muscle. Prolonged surface EMG biofeedback can facilitate improvement of function for behaviourally relevant tasks in each child’s natural environment.	[16]
Systematic review	Describing systematically the best available intervention evidence for children with cerebral palsy (CP).	Systematic review of systematic reviews.	The electronic feedback about muscle activity, to teach voluntary control, can improve muscle activation and active range of motion in an effective manner if combined with other treatments.	[17]

From the literature search and review in this section, it can be inferred that the orthotic treatments have been tested rather sparingly for MD, often in small groups of subjects or single case studies, thus reporting results that have not enough strength to be directly generalised into standard requirements or recommendations for their use. It seems that customisation [1] of orthoses could be the key for a better approach of the treatments reviewed, despite the clear necessity of broadening the quantity of patients enrolled, types of splints tested and the quality of studies designed [1, 17, 20]. The specific characteristics of the disease should be considered in the personalisation approach but it can be added that the understanding of the neurophysiological aspects could even drive the concept design of orthotic solutions.

### Neurophysiological considerations and therapeutic requirements for orthotics

The principal manifestations of the disease that will be considered here include variability in motor commands, overflow, hypertone and changes in sensorimotor integration. While these are typical traits of CD, they can often be observed in other MD’s.

### Variability

The higher-than-normal variability in the repeated execution of a motor task in dyskinetic cerebral palsy supports the interpretation of basal ganglia dysfunction as the lack of appropriate filtering of random motor schemes [3]. It has been shown that hyperkinetic and unwanted movements in upper extremities in children with dyskinetic CP are characterised by increased signal-dependent noise (SDN) of the motor system. The SDN theory states that noise in motor commands (e.g. spatial variability) tends to increase with the motor command’s magnitude (e.g. force, velocity). This means that children with dystonia require significantly slower movements to contain the increased motor variability if they want to achieve comparable precision to typically developing children. Even if the relationship between basal ganglia injury and increased signal dependent noise is not known, it could be inferred that, based on the model reported above, the Basal Ganglia disease might lead to decreased inhibition or perhaps even excitation of unwanted patterns, resulting in increased motor variability [6]. An increased sensitivity to the accuracy requirements of the task arises from an increase in the variability of movement with increasing movement speed. According to Sanger, the hypothesis of increased signal-dependent noise in dystonia provides a possible explanation on the basis of a decreased specificity in the selection of movements by the basal ganglia [21].

#### Lesson learnt and design implication

*The noise superimposed on the movement affects the outcome of motor task execution and causes variability and imprecision. The fact that there is a direct dependency between the noise, the movement amplitude and speed suggest that a solution, which reduces or at least contains the positioning noise could help patients achieve higher velocities and broader movement spans for a given precision.*

### Overflow

Overflow is a spread of motor activation to surrounding or distant muscles different from those typically recruited for the goal-directed action. The current hypothesis, expressed in the Mink model [22], is that dystonia results from incomplete suppression of competing motor patterns due to insufficient surround inhibition of competing motor patterns generators. This deficient surround inhibition may also lead to expansion of the facilitatory centre, which would lead to “overflow” contraction of adjacent muscles. Decreased efficacy of the surround inhibition with or without expansion of the centre causes inappropriate disinhibition of unwanted muscle activity.

#### Lesson learnt and design implication

*A contraction of unwanted muscles accompanying the activation of the specific muscles required for the execution of some task is the principal external expression of the dyskinetic overflow. This observation brings to different possible management approaches: (1) avoid to contract a muscle to prevent the undesired activation of adjacent muscles, (2) provide adequate forces to support the muscles involved in the execution of the desired motor task; in doing this, it is expected that a burden of voluntary control could be partially shifted to limiting the unwanted contractions, i.e. the overflow; (3) offer a direct constraint to control the movements not specifically involved for the execution of the action. While option (1) is inapplicable, lest the voluntary action is not achieved, options (2) and (3) could be implemented with different devices.*

### Hypertone

There is still a debate about the mechanisms by which hypertonia occurs in dystonia. It was previously thought to be the result of tonically co-contracting muscles that contribute to passive joint stiffness. This model is coherent with what is known about joint stiffening in normal posture. More recent studies suggest that reflexes may play an important role [6].

Co-contraction (the simultaneous contraction of antagonist muscles) is known to stabilise limb movements [23–25]. Co-contraction alters the biomechanical operating ranges of muscle and tendon by increasing both muscle-tendon stiffness (elasticity) and muscle damping (viscosity) [26–28]. The hypothesis that excessive tonic co-contraction is the main mechanism of stiffening in dystonia rests upon the above observations and is supported by the fact that co-contraction is present in adult focal dystonias [29]. On the other hand, this has not been tested in childhood generalised secondary dystonias. Furthermore, adult focal dystonia is often not associated with hypertonia at rest [29].

Looking at the considerations leading to hypertonia being linked to reflexes, there are several interesting results. One prominent feature of hypertonic dystonia is that a fixed posture may be maintained at a non-extreme joint angle. This is different from spasticity, in which fixed postures tend to occur at joint limits (maximal ankle or knee extension, or wrist flexion, for example). In the literature different theories have been suggested about the possible causes of hypertone and its manifestations. For example Kats et al. [30, 31] reported that a hypertonic limb with dystonia may not necessarily show involuntary muscle activation prior to movement. Since the characteristic activation does not exhibit a threshold velocity or angle leading to a “catch”, and since spinal mechanisms are presumably normal, it is likely that, if such dystonic reflex activation occurs, it occurs through a different mechanism from that of the monosynaptic stretch reflex [30, 31]. On the other hand, van Doornik [29] observed that during passive flexion and extension movements of the elbow, in 8 children with hypertonic arm dystonia due to dyskinetic cerebral palsy, there was always significant phasic electromyographic activity in the lengthening muscle, consistent with reflex activity. Based on the evidence that some children exhibit position-dependent activation, some exhibit velocity-dependent activation, and some exhibit a mixed pattern of activation, the same authors conclude that, the involuntary or reflex muscle activation in response to stretch, may also be a significant contributor to increased tone in hypertonic dystonia. Most of the subjects (7 out of 8) had little or no triceps activity at rest, which means that their biceps activity at rest was not a component of involuntary co-contraction of agonist and antagonist [29]. There have been attempts [32] to separate dystonic contributions from spastic ones in children affected by cerebral palsy. That was done using measures of overflow to other muscles (dystonic features) and force-velocity relationship (spastic features). The results showed that in spite of a “spastic” or “extrapyramidal” diagnosis, most children had a combination of both spasticity and dystonia. As demonstrated by the different, and not completely consonant, opinions stated by researchers over the years, and considering that, many of them suggested further investigations to shed more light on the nature of hypertone, a wise approach looking at the hypertone symptom in Childhood Dyskinesia is to point out the main features of this problem and a possible approach.

#### Lesson learnt and design implication

*The hypertone, which is thought to be a cause of joint stiffness during normal posture could be described both as co-contraction of opposing muscles, agonists and antagonists; or as a reflex-dependent response of a muscle. In the first case, as there is no dependency from position and speed, there can be a stable dystonic (ill-)posture, and the hyperactivation occurs along the entire range of motion: this picture leads to the possibility to exploit repositioning therapies, whose intensity will not impact on the level of muscular response. The reflex-dependent dystonia model, on the other hand, leads to a type of stiffness more similar to the spastic picture: it depends on spatial and/or temporal parameters (albeit without thresholds); this model suggests a different approach, which tends to obtain a more physiological posture by imparting forces that do not activate the stretch-reflex response, i.e. mild forces, producing gradual and slow postural evolution.*

### Proprioception and sensorimotor integration

Proprioception is so closely tied to the control of voluntary movement that impairments are more likely to be noticed as deficits in the performance of motor tasks than as impaired sensations [33]. Abnormalities in this sensorimotor integration underlie many hypokinetic and hyperkinetic movement disorders [34]. Increasing evidence of sensory system involvement makes it essential to consider the possible contribution to the pathophysiology of certain MD’s of changes in sensorimotor integration, that is to say, in the ability to use sensory information properly for assisting motor program execution [35]. Sensorimotor integration seems to play an important role in the disturbances of motor control (movement guide, muscle activation) typically seen in patients with Parkinson’s disease, Huntington’s disease and dystonia. Defective elaboration of sensory inputs is likely to be of particular clinical relevance to the development of focal forms of dystonia, where there are also deficits in visual-tactile-proprioceptive integration [34, 35]. It is known that for some specific forms of dystonia, such as cervical dystonias, writers’ cramp and focal dystonias, there are some specific actions that can be voluntarily done to reduce the symptoms connected to dystonia, such as ill postures, tremors etc. These actions, generally involving light-touch (not forceful constraining), are called *alleviating manoeuvres* (AM) or *sensory tricks* [36]. Such effects can be considered in the broader respect that sensory inputs can modify dystonia and they may influence dystonic contractions by changing the level of excitability in all the pathways, so as to alter the motor “focus” [37]. In the last years, new insights suggest that dystonia could originate from a lack of reliable sensory feedback regarding motor actions. In particular, dystonia could stem from distorted and excessive afferent inputs linked via reflexes to create abnormal motor outputs [6].

Even if the AM as such are specific for focal dystonias, the phenomena that rule this kind of effects (touch, sensory stimulation, proprioception) could be useful in the management of dystonic syndromes in general. In cervical dystonia sensory tricks seem to improve dystonia through an inhibitory effect on motor cortex excitability [38]. Proprioceptive stimulation could elicit AM [39], and their effectiveness is strongly dependent on the initial district position (e.g. head rotation angle) [40]. Furthermore the phenomenon of the sensory trick can take effect through a change in the level of fusimotor drive [37]. Actually, it has been shown that abnormal spindle function is present in focal dystonia [41–43]. This is consistent with Grünewald’s experiments of passive mobilisation and vibrotactile stimulation of the forearm. His results suggest that patients with focal dystonia can display abnormal perception, with an involvement of impaired muscle spindle function and in particular that there is an abnormal perception of motion, but not position [41]. These observations could be taken as a source of inspiration in designing approaches including sensory enhancement.

#### Lesson learnt and design implication

*Proprioceptive sensory input plays a crucial part in the generation and coordination of movements; peripheral sensory afferences are fundamental in planning and executing of the movement. Disruptions of sensory input integration are considered to be of clinical relevance to the development of focal forms of dystonia, and they are addressed more as linked to deficits in the performance of motor tasks than as merely sensory impairments* [32]. *In this respect, it could be a clever option to take these aspects into account, conceiving devices, that try to fulfil not only the biomechanical aims, but also offer proprioceptive and sensory inputs in general, especially as a feedback in response to a motor action.*

## Discussion of the available orthotic treatments in the light of neurophysiology and the current therapeutic trends

The *rigid orthoses* for Childhood Dyskinesia (or dystonia in general), exploiting the rigidity provided by the materials employed, are designed based on the idea to block and prevent the motion of the joints involved in either the residual voluntary movements or the involuntary ones, i.e. the dyskinetic actions [44]. Although the mechanisms of action of immobilisation are largely unknown, the paper by Priori [45] has postulated that removing all motor and sensory input to a limb, this could allow the cortical map to reset to the previous normal topography. Although the study is interesting for occupational focal upper-limb dystonia, it is not clear to us, how this could work for dyskinetic subjects in the developmental age, who were born without a normal topography. Furthermore, the idea to provoke sensory deprivation clashes with more abundant evidence that *appropriate* sensory input can alleviate many forms of dystonia. The different approach, implemented in the Egyptian research by El-Maksoud [46], shows how blocking one of the joints in the affected limb makes it easier for the dyskinetic children to control the other joints better. This method is interesting, because it suggests that *limiting the degrees of freedom to be governed, the system can re-distribute more effectively the control resources to carry out upper-limb motor tasks*.

Another observation regarding rigid orthoses and joint immobilisation has to do with the development of interface forces. On the one hand, limiting involuntary movements with fixed constraints produces discomfort due to the resulting pressures on the skin; on the other, the continuous isometric contraction of the muscles affected by hyperkinesia or overflow could generate hypertrophy and exacerbate the dominating presence of those muscles in determining posture. Furthermore, if the reflex-mediated interpretation [29] of hypertonic dystonia is true, the unyielding stretch produced by the rigid orthoses could directly worsen limb stiffness.

Discussing more in detail the results in the field of *soft orthoses* it could be inferred that, despite the larger compliance of the base material and adaptability of use, from a functional point of view, some of them do not provide well defined forces (Lycra vest), or the elastic strings (Adeli suit) and bands (Spiral suit) impart forces that are quasilinear: the more the elastic element is stretched, the higher will be the force (in order to control or support severe pathological postures, very strong elastic bands have often been applied with consequent discomfort and limitations in movement). So, these solutions could become ineffective for little elongations, i.e. around the resting position (non-stretched) or quite uncomfortable on the opposite extreme, i.e. end-range maximum stretching, together with straining in the psychological domain [17]. Furthermore even if these soft orthoses are tailored on the size of the patients, they are often reported to be uncomfortable to wear, especially the Lycra garment because of the donning and doffing problems [47]. The systematic reviews dealing with all of those soft suits also indicate the low quality of evidence related to their efficacy [17, 47, 48]. On the other hand, splints made of soft, semi-rigid or combined materials can be useful in supporting functional position during movement, containing the joint and giving proprioceptive information [19]. Bearing in mind all these aspects, it is evident that a careful *customisation of both shape and function* would be necessary to solve the negative aspects of these devices. What is interesting about the soft technologies, though, is that (1) *they allow for residual movements to be initiated and carried out voluntary by the patients*; (2) *they can provide some sensory stimulation that could be achieved in general through a wrapping stimulus, and in particular through the material response to an initiated motor action, or else by designing some parts in the orthosis ad hoc to this aim*.

Amongst the systems, which can *enhance stimulation*, there are the wearable solutions that provide biofeedback. Findings that come from the literature show how biofeedback can help some patients eliminate the co-contraction of the flexors and extensors and to quiet unnecessary motor contraction [44]. Some studies showed that muscle vibration may trigger focal hand dystonia, that can be partially relieved by muscle afferent block with lidocaine [35]. In contrast to that observation, vibrotactile systems for proprioceptive enhancement are being tested for the rehabilitation support in dystonic pictures, especially in those of focal dystonia [49] and in children with primary or secondary dystonia [50–52]. The principle of action of biofeedback is that it provides the subjects with instantaneous, reliable information on their performance, making covert physiological processes more manifest. The aim of biofeedback is to improve the efficacy of the rehabilitation treatment by allowing patients to adjust their movements. Additionally, biofeedback training is able to capture and maintain the subject’s attention and to allow the participant to exercise in immersive settings [52]. Electromyography-based biofeedback in particular has been reported for children with cerebral palsy [16]. Further research is warranted to determine the effectiveness of multiple simultaneous feedback modalities, such as tactile, visual, haptic and auditory, and to determine which particular motor deficits and types of dystonia are most influenced by these interventions [6]. While cognitively-intense definitions of biofeedback can be certainly useful in the framework of rehabilitation sessions, they may be less applicable in mixed rehabilitative-assistive treatments. In fact it is hard and tiring to maintain attention and focus for longer periods, and fatigue may arise; patients should be very collaborative and this is not always the case, especially dealing with children, in particular if they are very young or their conditions are severe, possibly including cognitive or behavioural deficits: they can even fail to completely understand the requests, or be frankly unable to try and control specific muscles due to excessive functional deficits in the neuromusculoskeletal system.

In this respect, a different less-cognitively-mediated definition of biofeedback could be more broadly applicable, i.e. one based on interoceptive sensation and proprioceptive feedback. For example, some patients with cervical dystonia were shown to benefit from specially-designed neck and head braces that provide a sensory input, by touching certain portions of the neck or head, in a fashion similar to a patient’s own sensory trick; they enable patients to maintain a desired head position [19]. Apart from this example referring to alleviating manoeuvres, also other *sensory (afferent) stimuli*, skilfully applied by people or objects are useful to facilitate movement: e.g. touch and pressure, traction and compression, stretch or limb elongation. In particular *the proprioceptive effect of muscles, contracting against a resistance* [53–56], could be considered: the sensation of a controlled resistance could enhance the feedback from the end effectors via natural afferent pathways and provide non-invasively an augmented guide for the central nervous system to manage execution errors. To achieve this target, rather than offering a constant stimulus independent from the motor action, orthoses could be designed *to respond to movement*, with appropriate force fields acting as sources of enhanced proprioceptive feedback. Light forces can be used to counteract dystonia [57], with the advantage that voluntary movement should not be hindered. Besides the intensity, also the *types of force* must be considered to control the variability, overflow, hypertone and enhance peripheral sensations. Some studies in the literature have investigated the relationship between the types of force applied to a muscle and the myoelectrical response of the muscle [58]. In particular, inertial loads prolong the durations of both agonist and antagonist contractions, increasing the areas of the rectified EMG bursts [59] and delaying the antagonist burst [60–62]. External viscous loads reduce the antagonist burst [63, 64], similar to the effect of intrinsic limb viscosity [65]. Elastic loads demand static torques to be maintained in order to hold some posture [64, 66]. Viscous loads also demonstrate the unusual property of causing changes in peak velocity without corresponding changes in movement time [67].

**Fig. 2:**
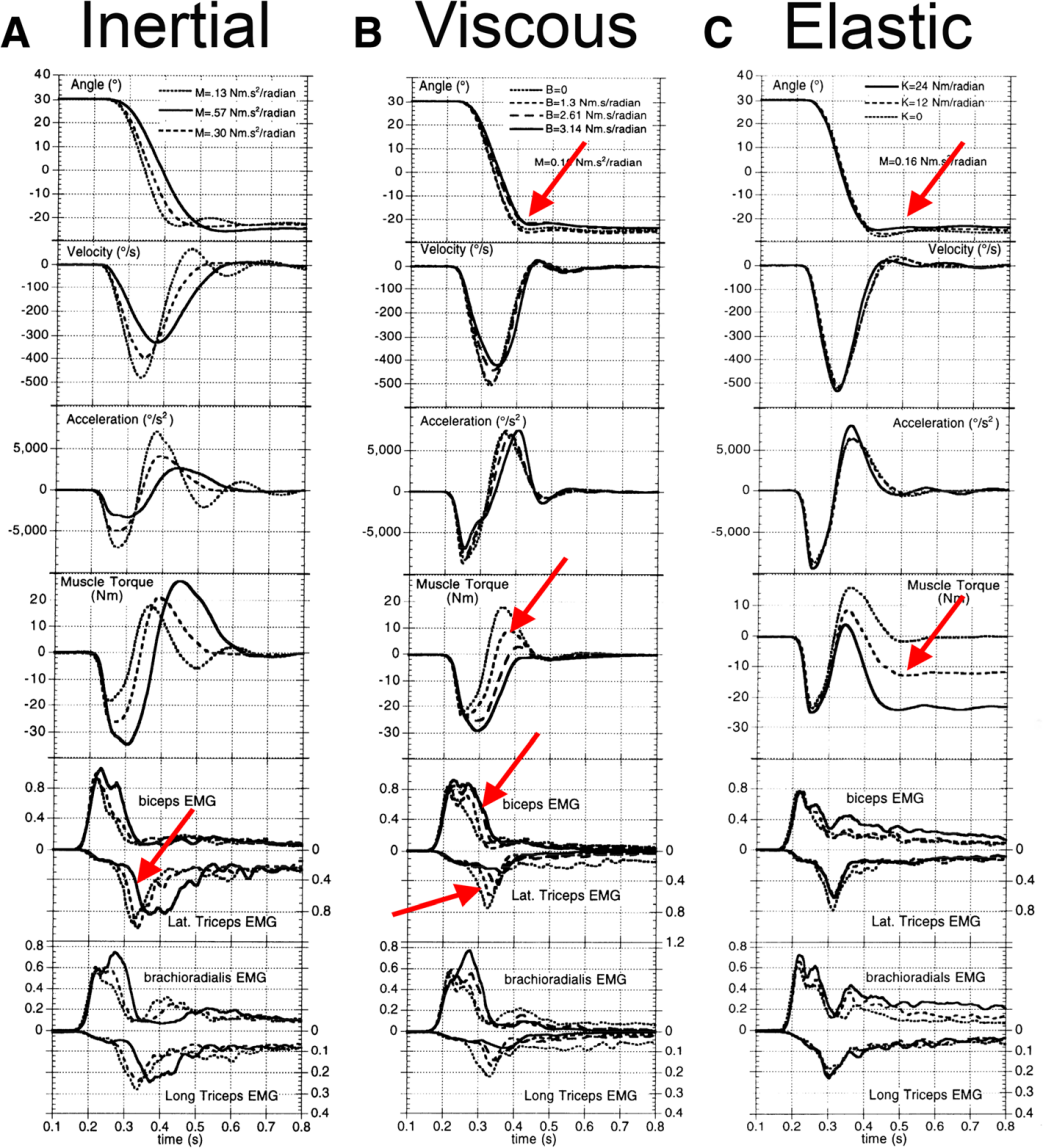
Effects of external forces on EMG. The figure, *from left to right respectively,* reports the kinematics (*first 3 graphs from the top*) and the dynamics (*last 3 graphs*) of the upper limb when different type of loads are applied: inertial, viscous and elastic forces. *Red arrows* underline the main variations in burst intensity: antagonist bursts increase for inertial loads, decreases with viscous loads, and decreases slightly with elastic loads (adapted from Gottlieb [67])

Agonist EMG bursts rise at a rate that is independent of the type or magnitude of the load. The areas of the antagonist bursts increase for inertial loads, decrease with viscous loads and decrease slightly with elastic loads. The areas of the agonist and antagonist bursts can be independently controlled by the subject, based on the torque requirements of an external load Fig. 2 [67].

All these findings suggest that a *personalised visco-elastic force field could be the best solution meeting the requirements connected to the orthotic management of Childhood Dyskinesia*, by offering a suitable tool for the rehabilitation and the support of daily activities.

## Conclusions: design principles for the development of new wearable orthotic systems

In the light of the above, the following final considerations are a practical basis to summarise the requirements that a wearable orthotic device to treat this movement disorder should possess.

Firstly, the orthosis should provide *dynamic postural control*: from a functional point of view, considering the hyperkinetic nature of the disease, this type of solution could be better than static and fixed ones, because the neutral position is allowed to vary in dependence of the fluctuations in the daily conditions of the subjects, and they are compliant towards involuntary movements. This choice is therefore expected to improve comfort, tolerability and decrease treatment times. When manufacturing the orthotic device the aim should be to re-define the angles of the limb joints, in order to facilitate the initiation and control of the movement chain. Since the posturing is dynamic, this corresponds to applying a bias force inducing a suitable *reference* (neutral) posture. In some cases the resting configuration of the device shall not directly correspond to the reference posture, but will tend to that, once a balance is found between the orthosis action and the force expressed by the patient. A dynamic and compliant device may be a better choice than a rigid one also because the potential voluntary actions are allowed. Consequently, a device exploiting the positive aspects of soft orthoses can provide a good balance between a rehabilitative and an assistive use.

Furthermore the orthosis should provide ***proprioceptive stimulation***. As it has emerged from the literature review, that it is important to have sensory information available to support motion. In particular, afferent stimuli from produced non-invasively could help improve and strengthen feedback control and support motor performance. In implementing a proprioceptive stimulation, it is possible to use *force fields that are generated by the orthosis in response to movement* by the patient. Paramount importance must be given to the choice of the *type of forces imparted*. By exploiting the characteristics of materials displaying ***visco-elastic damping behaviour***, it should be possible to stabilise the motion and improve the control of voluntary actions. Visco-elastic materials, in fact, can generate forces that do not stimulate an overshooting in the activation of the impaired muscular system, but are rather expected to reduce co-contractions, decrease tonic activities and thus contain hypertone and the negative phenomena of variability and overflow.

Another important aspect in designing future orthoses for Childhood Dyskinesia is *the level of the forces* imparted: keeping ***forces as low as possible*** may be a useful strategy to prevent the stretch-reflex response and to reduce the hypertone: that could help decrease the phasic and tonic responses of the muscles affected by the syndrome. The use of forces that can be considered mild with respect to the patient’s muscular strength is also appropriate, in that it may help safeguard the capability to carry out the voluntary actions.

While utilising a less-cognitively-mediated approach to proprioceptive enhancement, it would still be possible to implement additional *cognitively-intense biofeedback tools*. Considering the target of treatment is important, though: for instance, it could be too difficult for severe children to make the most of those features. In addition, *passive orthoses* can have the advantage of light weights and independence from a power supply, making them more comfortable and usable for the children, also during their daily activities.

The orthosis should also optimise the fit in order to improve comfort and function at the same time; *a light, comfortable and wearable device, that can be donned and doffed quite easily* is considered to be fundamental, especially for children in their developmental age.

Bearing in mind all the above, the *customisation aspects* are also key: aiming at a customised resting (target) posture for every different clinical picture (mainly hypertonic or mainly hyperkinetic, degrees of freedom mostly affected, degree and type of overflow, etc.), personalised viscoelastic forces, together with the fine design of contact surfaces anatomically adherent to the patient’s upper-limb shape all converge towards light, wearable and functional passive orthoses.

Our group is conducting pilot evaluations of orthoses made according to the present guidelines, and exploiting the properties of pseudoelastic Nitinol alloy. Some very preliminary results have been published [68], addressing feasibility, acceptance and clinical effects.

Considering the variety of existing MD with hyperkinetic and/or hypertonic traits, often combined with sensory deficits, it is expected that the presented approach could be extended to different diseases with similar components, affecting both children and the adult, such as genetic, metabolic and degenerative forms.

## Abbreviations

MD: Movement Disorders
CD: Childhood Dyskinesia
CP: Cerebral palsy
NDT: Neuro-developmental therapy
SDN: Signal-dependent noise
AM: Alleviating manoeuvres
EMG: Electromyography
